# Individual and neighborhood-level socioeconomic characteristics in relation to smoking prevalence among black and white adults in the *S*outheastern United States: a cross-sectional study

**DOI:** 10.1186/1471-2458-11-877

**Published:** 2011-11-21

**Authors:** Sarah S Cohen, Jennifer S Sonderman, Michael T Mumma, Lisa B Signorello, William J Blot

**Affiliations:** 1 International Epidemiology Institute, 1455 Research Blvd, Suite 550, Rockville, MD 20850, USA; 2 Division of Epidemiology, Department of Medicine, Vanderbilt University Medical Center and Vanderbilt-Ingram Cancer Center, 2525 West End Ave, Nashville, TN 37203, USA

**Keywords:** Cigarette smoking, Socioeconomic status, Race, Residence characteristics

## Abstract

**Background:**

Low individual-level socioeconomic status (SES) is associated with higher prevalence of cigarette smoking. Recent work has examined whether neighborhood-level SES may affect smoking behavior independently from individual-level measures. However, few comparisons of neighborhood-level effects on smoking by race and gender are available.

**Methods:**

Cross-sectional data from adults age 40-79 enrolled in the Southern Community Cohort Study from 2002-2009 (19, 561 black males; 27, 412 black females; 6, 231 white males; 11, 756 white females) were used in Robust Poisson regression models to estimate prevalence ratios (PRs) and 95% confidence intervals (CI) for current smoking in relation to individual-level SES characteristics obtained via interview and neighborhood-level SES characteristics represented by demographic measures from US Census block groups matched to participant home addresses.

**Results:**

Several neighborhood-level SES characteristics were modestly associated with increased smoking after adjustment for individual-level factors including lower percentage of adults with a college education and lower percentage of owner-occupied households among blacks but not whites; lower percentage of households with interest, dividends, or net rental income among white males; and lower percentage of employed adults among black females.

**Conclusions:**

Lower neighborhood-level SES is associated with increased smoking suggesting that cessation programs may benefit from targeting higher-risk neighborhoods as well as individuals.

## Background

Cigarette smoking is a major risk factor for a multitude of diseases [1], and despite declines in smoking in recent decades, an estimated 24% of men and 18% of women in the United States were smokers in 2009 [2]. Low individual socioeconomic status (SES) is strongly associated with increased smoking prevalence across race and gender lines [3], and recent work has begun to examine whether socioeconomic characteristics of the neighborhood in which a person resides influence smoking behavior independently from individual-level SES [4-11]. Several plausible mechanisms have been suggested to explain how neighborhood-level factors might affect smoking behavior including the influence of neighborhood cultural or normative standards [4], geographic distribution of tobacco advertising [12], and psychosocial stress related to disadvantaged neighborhood settings [5]. If neighborhood SES characteristics affect smoking behaviors above and beyond the influences of individual SES through these mechanisms or other pathways yet to be determined, novel public health interventions to reduce smoking initiation and encourage smoking cessation may be developed to target high-risk neighborhoods as well as individuals. However, before such interventions can be developed and appropriately tailored, research is needed to determine whether differences exist in the effects of neighborhood characteristics across race and gender groups. To date, however, most studies that have examined neighborhood SES in relation to smoking have had limitations regarding sample composition that have prevented robust comparisons of associations between neighborhood-level characteristics and smoking by race and gender. For example, in the United States, various measures of lower neighborhood-level SES have been associated with increased smoking prevalence in a study of young black and white adults (age 18-30) [5], in small study populations in North Carolina [7] and Illinois [4], in a large national sample of black women [8], and in participants residing in four communities (one of which included black participants) in the Atherosclerosis Risk in Communities (ARIC) study [13]. To improve upon the limited comparisons across race and gender groups in these studies, we examined associations between current cigarette smoking and both individual-level and neighborhood-level characteristics in a large group of black and white adults age 40-79 living in twelve states in the southeastern US.

## Methods

### Study population and data collection

**Figure 1:**
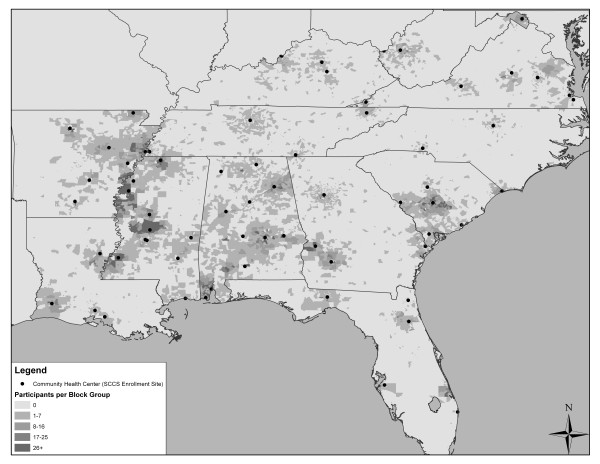
Location of Community Health Centers for participant enrollment into the Southern Community Cohort Study as well as distribution of 64, 960 participants residing in 10, 168 block groups from the 2000 United States Census

The Southern Community Cohort Study (SCCS) is an ongoing prospective cohort study designed to investigate health disparities in understudied populations [14,15]. Institutional Review Boards at Vanderbilt University and Meharry Medical College approved the study and study participants provided informed consent at the time of enrollment. Cohort enrollment took place at 71 community health centers (CHCs), institutions that provide health services primarily to low income and uninsured persons [16], in twelve southeastern states (Figure 1). SCCS eligibility requirements included being age 40-79 years, English-speaking, and not having been under treatment for cancer in the past 12 months. Nearly 73, 000 participants were recruited via CHCs from 2002 to 2009.

Participants were administered an in-person baseline interview by a trained study interviewer at enrollment. The computer-assisted personal interview contained questions about demographic, medical, familial, lifestyle and other participant characteristics (the questionnaire is available online [17]). Individuals were classified as current smokers if they answered yes to both of the interview questions "Have you smoked at least 100 cigarettes in your entire life?" and "Do you smoke cigarettes now?" Smoking self-report was validated against serum cotinine in 337 SCCS participants who reported no exposure to passive tobacco smoke, and misclassification of smoking status was found to be very low (2% for current smokers, 6% for former smokers, and 4% for never smokers based on a serum cotinine cut-off of 15 ng/mL) (personal communication, LB Signorello).

SES characteristics of the individual study participants, termed "individual-level characteristics, " were obtained from the baseline interview and included annual household income, educational attainment, marital status, and current employment status. Participants provided their home address at the time of study enrollment as well as their duration of residency. The home addresses were geocoded using the ArcView 9.3 Desktop Address Locator (ESRI, Redlands, CA) with ESRI's Streetmap USA shapefiles as the reference database [18]. Geocoding of addresses that failed using ArcView was attempted first with the Tiger/Line 2008 shapefiles as reference [19] and then with an online geocoding vendor. 88.6% of addresses were geocoded to the street address level, 1.7% were geocoded using a ZIP+2 or ZIP +4 centroid, 9.6% were geocoded using a delivery-weighted 5-digit ZIP code centroid, and only 0.1% completely failed to geocode. Geocoded home addresses were linked to data from the 2000 US Census at the block group level. Block groups typically include between 600 and 3, 000 people with a target size of 1, 500 and are the lowest level of the census geographic hierarchy for which demographic data are released by the US Census Bureau [20]. Nine SES-related measures, termed "neighborhood-level characteristics", were examined for each block group for these analyses and are listed in Table 1.

**Table 1:** Individual-level and neighborhood-level characteristics for 64, 960 participants enrolled in the Southern Community Cohort Study via Community Health Centers, 2002-2009

	Black				White			
	
	Male		Female		Male		Female	
	
	N	(%)	N	(%)	N	(%)	N	(%)
Individual-level variables^a^								

Total number of persons	19, 561	30.1	27, 412	42.2	6, 231	9.6	11, 756	18.1

Number of census block groups	4, 825		5, 594		3, 433		4, 836	

Years lived in current home (mean)	9.3		10.4		8.1		9.3	

Age at enrollment (mean)	50.4		51.6		52.5		53.5	

Household income								

< $15, 000	12, 354	63.2	16, 859	61.5	3, 565	57.2	6, 582	56.0

$15, 000-$24, 999	4, 281	21.9	6, 411	23.4	1, 310	21.0	2, 427	20.6

$25, 000-$49, 999	2, 221	11.4	3, 156	11.5	878	14.1	1, 652	14.1

> $50, 000	705	3.6	986	3.6	478	7.7	1, 095	9.3

Education (years)								

< 9	1, 820	9.3	2, 026	7.4	708	11.4	981	8.3

9 - < 12	5, 060	25.9	6, 494	23.7	1, 200	19.3	2, 287	19.5

12 - < 16	11, 393	58.2	16, 414	59.9	3, 635	58.3	7, 155	60.9

≥16	1, 288	6.6	2, 478	9.0	688	11.0	1, 333	11.3

Marital status								

Married	5, 736	29.3	7, 312	26.7	2, 587	41.5	5, 097	43.4

Separated/Divorced	6, 685	34.2	9, 487	34.6	2, 270	36.4	4, 144	35.3

Widowed	733	3.7	3, 883	14.2	259	4.2	1, 606	13.7

Single/Never married	6, 407	32.8	6, 730	24.6	1, 115	17.9	909	7.7

Currently working								

Yes	7, 220	36.9	10, 706	39.1	1, 948	31.3	3, 959	33.7

No	12, 341	63.1	16, 706	60.9	4, 283	68.7	7, 797	66.3

Cigarette smoking status								

Current	11, 609	59.3	9, 220	33.6	3, 258	52.3	4, 623	39.3

Former	3, 786	19.4	5, 258	19.2	1, 758	28.2	2, 890	24.6

Never	4, 166	21.3	12, 934	47.2	1, 215	19.5	4, 243	36.1

Neighborhood-level variables^b^								

Percent poverty								

< 10%	2, 027	10.4	2, 969	10.8	1, 747	28.0	3, 618	30.8

10 - < 20%	3, 659	18.7	5, 775	21.1	2, 143	34.4	4, 409	37.5

20 - < 50%	11, 204	57.3	15, 137	55.2	2, 184	35.1	3, 549	30.2

> 50%	2, 671	13.7	3, 531	12.9	157	2.5	180	1.5

Household income								

< $18, 879	6, 442	32.9	8, 128	29.7	722	11.6	896	7.6

$18, 879 - < $26, 094	5, 389	27.5	7, 522	27.4	1, 228	19.7	2, 152	18.3

$26, 094 - < $34, 583	4, 124	21.1	6, 324	23.1	1, 942	31.2	3, 835	32.6

> $34, 583	3, 606	18.4	5, 438	19.8	2, 339	37.5	4, 873	41.5

Percent adults with ≥ HS education^c^								

< 57.8%	6, 009	30.7	7, 769	28.3	966	15.5	1, 486	12.6

57.8% - < 67.5%	5, 186	26.5	7, 472	27.3	1, 232	19.8	2, 363	20.1

67.5% - < 77.3%	4, 408	22.5	6, 433	23.5	1, 812	29.1	3, 585	30.5

> 77.3%	3, 958	20.2	5, 738	20.9	2, 221	35.6	4, 322	36.8

Percent adults with ≥ college graduation^c^								

< 5.8%	5, 423	27.7	7, 136	26.0	1, 339	21.5	2, 340	19.9

5.8 - < 10.3%	4, 963	25.4	6, 828	24.9	1, 466	23.5	2, 987	25.4

10.3 - < 17.1%	4, 450	22.7	7, 003	25.5	1, 572	25.2	3, 195	27.2

> 17.1%	4, 725	24.2	6, 445	23.5	1, 854	29.8	3, 234	27.5

Percent households owner occupied								

< 43%	6, 304	32.2	7, 659	27.9	1, 025	16.5	1, 243	10.6

43 - < 63%	5, 408	27.6	7, 298	26.6	1, 329	21.3	2, 237	19.0

63 - < 80%	4, 505	23.0	6, 840	25.0	1, 566	25.1	3, 311	28.2

> 80%	3, 344	17.1	5, 615	20.5	2, 311	37.1	4, 965	42.2

Median owner-occupied household value								

< $44, 300	5, 570	28.5	7, 713	28.1	1, 049	16.8	1, 875	15.9

$44, 300 - < $57, 300	5, 187	26.5	7, 593	27.7	1, 136	18.2	2, 177	18.5

$57, 300 - < $77, 400	4, 598	23.5	6, 589	24.0	1, 768	28.4	3, 438	29.2

> $77, 400	4, 206	21.5	5, 517	20.1	2, 278	36.6	4, 266	36.3

Percent households with interest, dividends, or net rental income								

< 8.0%	6, 606	33.8	8, 459	30.9	541	8.7	622	5.3

8.0 - < 14.7%	5, 714	29.2	7, 954	29.0	958	15.4	1, 623	13.8

14.7 - < 24.1%	4, 104	21.0	6, 483	23.7	1, 880	30.2	3, 763	32.0

> 24.1%	3, 137	16.0	4, 516	16.5	2, 852	45.8	5, 748	48.9

Percent employed								

< 49.5%	5, 990	30.6	7, 525	27.5	993	15.9	1, 712	14.6

49.5% - < 56.9%	5, 142	26.3	7, 459	27.2	1, 265	20.3	2, 389	20.3

56.9% - < 64.1%	4, 352	22.2	6, 384	23.3	1, 835	29.4	3, 673	31.2

> 64.1%	4, 077	20.8	6, 044	22.0	2, 138	34.3	3, 982	33.9

Percent employed in management, professional, and related occupations								

< 15.1%	5, 654	28.9	7, 346	26.8	1, 220	19.6	2, 043	17.4

15.1 - < 21.2%	4, 804	24.6	7, 060	25.8	1, 480	23.8	2, 873	24.4

21.2 - < 28.3%	4, 729	24.2	6, 858	25.0	1, 544	24.8	3, 108	26.4

> 28.3%	4, 374	22.4	6, 148	22.4	1, 987	31.9	3, 732	31.7

Neighborhood advantage summary score								

< -4.2	5, 074	25.9	6, 460	23.6	612	9.8	850	7.2

-4.2 - < -1.9	4, 472	22.9	6, 194	22.6	836	13.4	1, 486	12.6

-1.9 - < 0.4	3, 709	19.0	5, 628	20.5	1, 183	19.0	2, 472	21.0

0.4 - < 3.7	3, 168	16.2	4, 895	17.9	1, 656	26.6	3, 273	27.8

> 3.7	3, 138	16.0	4, 235	15.4	1, 944	31.2	3, 675	31.3

^a^Individual-level characteristics were obtained from the baseline SCCS interview

^b^Neighborhood-level characteristics were obtained from the 2000 US census for block groups. All census measures (except percent poverty and neighborhood advantage summary score) were categorized into quartiles based on the distribution of the entire sample

^c^Among adults age 25 and older

### Statistical methods

All neighborhood-level measures were categorized into quartiles based on the distribution in the entire study sample, except block group percent poverty which was categorized as < 10%, 10-19%, 20-49%, and ≥ 50% for comparability to existing literature. A neighborhood advantage summary score was calculated as described by Diez Roux et al. [21] by summing z-scores (which reflect the deviation of each individual value from the overall mean in units of standard deviations) calculated using the distributions of variables in the entire study sample for the following variables: log median household income; percentage households receiving interest, dividend, or net rental income; log median value of housing units; percentage adults who completed high school; percentage adults who completed college; and percentage persons in executive, managerial, or professional specialty occupations. The range of the neighborhood advantage summary score was-12.7 to 28.7 with increasing score representing increasing neighborhood advantage. Quintiles for the neighborhood advantage summary score were created based on the entire study population distribution.

Cross-tabulations by race and sex were calculated for individual-level income and education by the neighborhood advantage summary score to assess the degree of overlap of participants between individual-level and neighborhood-level measures of SES.

The primary outcome was a dichotomous measure of current cigarette smoking (smoker versus non-smoker) as determined from the baseline interview. Poisson regression models were used to calculate prevalence ratios (PR) and 95% confidence intervals (CI) for current smoking. Prevalence ratios were selected as the measure of association instead of odds ratios because the outcome of current smoking was common (34-59%) in all race-sex groups. All models were adjusted for participant age as well as duration of residency at the current address because long-time residents were thought to be potentially more influenced by neighborhood characteristics than short-term residents. Smoking was first examined in relation to individual SES characteristics. Next we were interested in estimating the average relationship between neighborhood characteristics and smoking (i.e., not the conditional effects for specific neighborhoods). Exploratory data analysis revealed limited variation in neighborhood-specific smoking PRs, estimated by a random intercept for neighborhood, for all race and gender strata (standard deviation [SD] of intercept = 0.001 for all groups, *p *≥ 0.47) except black females (SD = 0.22, *P *< 0.001), indicating that little correction of standard errors was necessary for valid inference regarding neighborhood-level characteristics. We thus constructed marginal Poisson models using generalized estimating equations (GEE) to calculate population-average PRs with robust sandwich estimators to account for the small amount of clustering within neighborhoods, particularly for black females [22-25]. We computed the Score Test/Lagrange Multiplier Test for nested models to examine interactions with race/gender and neighborhood SES. This test had a p-value of 0.001 for the interaction between race and neighborhood advantage summary score, and *p *= 0.036 for the interaction between gender and neighborhood advantage summary score, and thus all models were stratified by race and gender. All analyses were conducted using SAS/STAT software Version 9.2 of the SAS System for Windows (SAS Institute Inc., Cary, NC).

## Results

Of the 72, 615 participants enrolled in the SCCS via CHCs from 2002-2009, 64, 960 (19, 561 black males, 27, 412 black females, 6, 231 white males, and 11, 756 white females) were included in the final analysis. Exclusions included 2, 953 (4.1%) participants who reported their race as being other than 'White' or 'Black/African American'; 139 (0.2%) with missing information on cigarette smoking; 1, 854 (2.6%) with missing information on individual-level characteristics; 82 (0.1%) whose address could not be geocoded; 344 (0.5%) whose address was outside of the 12-state enrollment area; 1, 780 (2.5%) who resided in such small block groups that the area measures were deemed to be unreliable (population < 300, housing units < 30, or > 33% of individuals living in group quarters); and 503 (0.7%) missing block group owner-occupied housing status.

The location and participant count of the 10, 168 block groups for the 64, 960 SCCS participants' home addresses at SCCS enrollment are shown in Figure 1. A mean of 6.4 participants resided in each block group (range 1-245). Individual-level household income and educational attainment were generally low among both blacks and whites (Table 1). As expected based on the large proportion of low-income participants, smoking prevalences were high among cohort members, and males were more likely to be current smokers than females. In contrast to the relatively similar distribution of individual-level education and income between the race groups, large differences were observed in the distribution of neighborhood-level SES characteristics with blacks being much more likely than whites to reside in block groups of lower SES (Table 1).

Cross-tabulations of participants across individual-level income and education and the neighborhood advantage summary score showed that, as expected, large numbers of participants with low individual income and education lived in low advantage neighborhoods, and similarly, large numbers of individuals of high individual income and education lived in high advantage neighborhoods (Table 2). Notably, however, meaningful numbers of participants were found across all categories of individual- by neighborhood-level SES. Differences by race were evident in the cross-tabulations; among individuals with household income < $15, 000/year, 13.4% and 11.9% of black males and females, respectively, lived in the most advantaged neighborhoods while 26.7% of white males and 26.0% of white females resided in the highest advantaged neighborhoods.

**Table 2:** Cross-tabulation of Individual-level income and education by neighborhood-level Neighborhood Advantage Summary score among participants enrolled in the Southern Community Cohort Study

Neighborhood advantage summary score (neighborhood-level)					
	**Quintile 1 < -4.2**	**Quintile 2 -4.2 - -1.9**	**Quintile 3 -1.9 - 0.4**	**Quintile 4 -0.4 - 3.7**	**Quintile 5 > 3.7**

	**%**	**%**	**%**	**%**	**%**

Black males					

Individual-level household income					

< $15, 000	29.9	23.5	18.5	14.6	13.4

$15, 000-$24, 999	21.8	23.6	19.9	17.9	16.8

$25, 000-$49, 999	16.6	20.2	20.9	20.1	22.2

≥$50, 000	10.1	15.0	14.9	21.4	38.6

Individual-level education (years)					

< 9 years	31.9	24.3	19.6	13.5	10.7

9- < 12 years	29.7	23.5	18.8	15.8	12.3

12- < 16	24.2	22.9	19.3	16.6	17.0

≥16	18.2	17.9	15.7	18.2	30.0

Black females					

Individual-level household income					

< $15, 000	27.9	24.1	20.2	15.9	11.9

$15, 000-$24, 999	19.9	22.1	21.5	20.2	16.3

$25, 000-$49, 999	13.3	18.4	21.5	22.4	24.4

≥$50, 000	6.5	13.3	16.3	21.3	42.6

Individual-level education (years)					

< 9 years	29.6	24.5	22.3	14.2	9.5

9- < 12 years	30.3	23.7	20.4	14.9	10.7

12- < 16	21.6	22.9	20.4	19.1	16.0

≥16	14.0	16.2	20.5	20.3	28.9

White males					

Individual-level household income					

< $15, 000	12.5	14.8	19.6	26.4	26.7

$15, 000-$24, 999	9.2	13.1	19.5	27.0	31.1

$25, 000-$49, 999	4.3	10.6	17.5	29.7	37.8

≥$50, 000	1.5	8.8	15.9	21.1	52.7

Individual-level Education (years)					

< 9 years	12.9	18.6	25.0	25.4	18.1

9- < 12 years	13.2	14.8	20.6	27.5	24.0

12- < 16	8.9	13.1	18.4	27.4	32.1

≥16	5.7	7.3	13.1	21.7	52.3

White females					

Individual-level household income					

< $15, 000	9.5	14.1	21.8	28.5	26.0

$15, 000-$24, 999	6.7	12.3	22.6	28.0	30.4

$25, 000-$49, 999	2.7	10.0	20.0	29.0	38.2

≥$50, 000	1.5	8.2	14.3	21.8	54.2

Individual-level education (years)					

< 9 years	12.1	18.1	22.1	28.2	19.4

9- < 12 years	10.5	15.2	22.8	30.4	21.1

12- < 16	6.2	12.1	21.6	28.0	32.2

≥16	3.6	7.3	14.3	22.4	52.4

In regression models including only individual-level measures, both individual-level income and education were strongly associated with smoking in each race and sex group. In the lowest v. highest category of household income, PRs (95% CIs) for smoking were 1.71 (1.52-1.91) for black males, 1.78 (1.53-2.08) for black females, 1.63 (1.39-1.91) for white males, and 2.06 (1.78-2.38) for white females. Similarly for categories of education comparing lowest to highest levels, PRs (95% CIs) for smoking were 1.14 (1.05-1.23) for black males, 1.30 (1.17-1.45) for black females, 1.28 (1.13-1.45) for white males, and 1.62 (1.44-1.83) for white females.

Next, each neighborhood-level characteristic was examined individually in relation to current smoking in robust Poisson regression models accounting for within-neighborhood correlation with adjustment for individual-level characteristics (Table 3). Lower quartiles of neighborhood-level household income, percentage of adults with a high school education, percentage of owner-occupied housing units, and percentage of households earning interest, dividends, or rental income as well as higher quartiles of percentage in poverty were all associated with increased smoking in each sex and race group except white women. Lower quartiles of percentage of adults with a college education and percentage of adults employed in professional occupations were both associated with increased prevalence of smoking only among blacks, and the effects were strongest among black women. Decreasing neighborhood advantage summary score was associated with increased prevalence of smoking most clearly in black women with evidence of a similar but more modest trend being apparent for the other race and sex groups (Table 3).

**Table 3:** Prevalence ratios for current cigarette smoking according to categories of neighborhood-level SES characteristics from race- and sex-stratified robust Poisson regression models^a^

	Black males		Black females		White males		White females	
	
	PR	95% CI	PR	95% CI	PR	95% CI	PR	95% CI
Percent poverty								

< 10%	0.92	(0.87-0.97)	0.78	(0.73-0.84)	0.81	(0.74-0.89)	0.99	(0.87-1.13)

10 - < 20%	0.95	(0.91-0.99)	0.84	(0.79-0.90)	0.80	(0.73-0.88)	1.01	(0.89-1.14)

20 - < 50%	0.99	(0.95-1.02)	0.91	(0.86-0.96)	0.82	(0.75-0.90)	1.00	(0.88-1.13)

≥ 50%	1.0		1.0		1.0		1.0	

0zHousehold income								

< $18, 879	1.08	(1.04-1.13)	1.24	(1.17-1.31)	1.10	(1.04-1.18)	0.99	(0.91-1.07)

$18, 879 - < $26, 094	1.05	(1.01-1.10)	1.18	(1.12-1.26)	0.96	(0.90-1.03)	1.00	(0.94-1.06)

$26, 094 - < $34, 583	1.02	(0.98-1.07)	1.07	(1.00-1.14)	1.00	(0.94-1.05)	1.00	(0.96-1.06)

≥$34, 583	1.0		1.0		1.0		1.0	

Percent adults with ≥ HS education^b^								

< 57.8%	1.05	(1.01-1.09)	1.20	(1.13-1.26)	1.06	(1.00-1.13)	1.03	(0.97-1.11)

57.8% - < 67.5%	1.01	(0.97-1.05)	1.12	(1.06-1.18)	0.95	(0.89-1.02)	1.01	(0.95-1.07)

67.5% - < 77.3%	1.02	(0.98-1.06)	1.06	(1.00-1.12)	1.01	(0.96-1.07)	1.03	(0.98-1.09)

≥77.3%	1.0		1.0		1.0		1.0	

Percent adults with ≥ college graduation^b^								

< 5.8%	1.05	(1.01-1.08)	1.22	(1.15-1.28)	1.02	(0.96-1.08)	1.04	(0.98-1.11)

5.8 - < 10.3%	1.05	(1.01-1.08)	1.11	(1.05-1.18)	0.97	(0.91-1.04)	1.03	(0.97-1.09)

10.3 - < 17.1%	1.01	(0.97-1.05)	1.04	(0.98-1.10)	0.97	(0.91-1.03)	1.03	(0.97-1.10)

≥17.1%	1.0		1.0		1.0		1.0	

Percent households owner occupied								

< 43%	1.20	(1.15-1.25)	1.41	(1.33-1.49)	1.11	(1.05-1.18)	1.01	(0.94-1.08)

43 - < 63%	1.13	(1.08-1.18)	1.25	(1.18-1.33)	1.04	(0.98-1.11)	1.03	(0.98-1.09)

63 - < 80%	1.10	(1.04-1.15)	1.17	(1.10-1.24)	0.97	(0.91-1.03)	0.97	(0.92-1.02)

≥80%	1.0		1.0		1.0		1.0	

Median owner-occupied household value								

< $44, 300	0.97	(0.94-1.01)	1.04	(0.99-1.10)	0.99	(0.93-1.06)	1.05	(0.99-1.12)

$44, 300 - < $57, 300	0.98	(0.95-1.02)	1.04	(0.98-1.10)	0.99	(0.93-1.06)	0.98	(0.92-1.05)

$57, 300 - < $77, 400	1.00	(0.96-1.04)	1.06	(1.00-1.12)	1.02	(0.96-1.08)	1.05	(1.00-1.11)

≥$77, 400	1.0		1.0		1.0		1.0	

Percent households with interest, dividends, or net rental income								

< 8.0%	1.08	(1.04-1.12)	1.23	(1.16-1.31)	1.17	(1.10-1.24)	1.03	(0.96-1.12)

8.0 - < 14.7%	1.07	(1.03-1.12)	1.13	(1.07-1.21)	1.07	(1.01-1.13)	1.02	(0.95-1.09)

14.7 - < 24.1%	1.03	(0.98-1.07)	1.04	(0.98-1.11)	1.01	(0.95-1.07)	1.04	(0.99-1.09)

≥24.1%	1.0		1.0		1.0		1.0	

Percent Employed								

< 49.5%	1.03	(0.99-1.06)	1.14	(1.08-1.20)	1.00	(0.94-1.07)	0.98	(0.92-1.05)

49.5% - < 56.9%	0.99	(0.96-1.03)	1.10	(1.04-1.17)	0.96	(0.90-1.02)	0.99	(0.93-1.05)

56.9% - < 64.1%	0.99	(0.95-1.03)	1.06	(1.00-1.12)	0.97	(0.92-1.03)	1.04	(0.99-1.10)

≥64.1%	1.0		1.0		1.0		1.0	

Percent employed in management, professional, and related occupations								

< 15.1%	1.05	(1.01-1.08)	1.24	(1.17-1.30)	1.02	(0.96-1.09)	1.04	(0.98-1.11)

15.1 - < 21.2%	1.05	(1.01-1.09)	1.09	(1.03-1.15)	1.03	(0.97-1.10)	1.06	(1.00-1.13)

21.2 - < 28.3%	1.00	(0.96-1.04)	1.03	(0.98-1.10)	0.99	(0.93-1.06)	0.99	(0.93-1.05)

≥28.3%	1.0		1.0		1.0		1.0	

Neighborhood advantage summary score								

< -4.2	1.05	(1.01-1.09)	1.24	(1.17-1.32)	1.10	(1.03-1.18)	1.07	(0.99-1.16)

-4.2 - < -1.9	1.05	(1.00-1.09)	1.17	(1.10-1.25)	1.00	(0.93-1.08)	1.04	(0.97-1.11)

-1.9 - < 0.4	0.99	(0.94-1.03)	1.03	(0.96-1.10)	0.96	(0.89-1.03)	1.01	(0.95-1.08)

0.4 - < 3.7	1.01	(0.97-1.06)	1.05	(0.98-1.12)	1.05	(0.99-1.11)	1.06	(1.00-1.12)

≥3.7	1.0		1.0		1.0		1.0	

^a^Each neighborhood-level characteristic was examined individually in relation to current cigarette smoking. All models include adjustment for individual-level income, education, marital status, and currently working (all categories as in Table 1) as well as participant age and duration of residency in the current home as reported during the baseline interview

^b^Among adults age 25 and older

Models examining the summary neighborhood advantage score in relation to smoking were further stratified by individual-level household income (< $25, 000/year versus > $25, 000/year) (data not shown). There was some indication that individual-level household income modified the association between smoking and neighborhood advantage score. Decreasing neighborhood advantage was associated with increased smoking mainly among those in the higher individual-level income group. PRs (95% CI) for current smoking in the lowest v. highest quintile of neighborhood advantage were 1.30 (1.13-1.50) for those with individual-level income > $25, 000/year compared to 1.02 (0.98-1.06) for those with income < $25, 000/year among black males, 1.41 (1.17-1.70) versus 1.22 (1.15-1.3) in black females, 1.19 (0.89-1.58) versus 1.10 (1.02-1.17) in white males, and 1.35 (1.000-1.81) versus 1.05 (0.97-1.14) in white females.

Table 4 shows prevalence ratios from a single robust Poisson regression model for each race and gender group that included all individual-level characteristics as well as all neighborhood-level characteristics (except for the summary z-score which was highly correlated with its individual components, and percent living in poverty and percentage in professional occupations which had high correlations, *ρ *> 0.8, with other neighborhood-level measures). Individual-level measures of household income, education, employment status, and marital status were associated with current smoking in these models and the magnitude of the associations was essentially unchanged from models including only individual-level characteristics. In these models, many of the associations between neighborhood-level characteristics and smoking seen in models including each neighborhood-level characteristic individually were attenuated. Among the neighborhood-level characteristics, the lowest quartiles of percentage with a college education and percentage of owner-occupied households were each associated with increased smoking among black men and women but not whites. Unexpectedly, increasing quartiles of median household value were associated with increased risk of smoking in blacks. Percentage of households with interest, dividends, or net rental income was associated with smoking in white males only while percent employed was associated with smoking only in black females.

**Table 4:** Prevalence ratios for current cigarette smoking according to categories of individual-level and neighborhood-level SES characteristics from race- and sex-stratified multivariate robust Poisson regression models^a^

	Black males		Black females		White males		White females	
	
	PR	95% CI	PR	95% CI	PR	95% CI	PR	95% CI
Individual-level variables								

Household income								

< $15, 000	1.67	(1.49-1.88)	1.68	(1.43-1.98)	1.60	(1.36-1.87)	2.04	(1.76-2.36)

$15, 000-$25, 000	1.50	(1.33-1.68)	1.48	(1.26-1.73)	1.52	(1.29-1.78)	1.86	(1.60-2.15)

$25, 000-$50, 000	1.31	(1.16-1.48)	1.24	(1.05-1.47)	1.35	(1.14-1.59)	1.45	(1.24-1.69)

≥$50, 000	1.00		1.00		1.00		1.00	

Education (years)								

< 9	1.14	(1.06-1.24)	1.27	(1.14-1.41)	1.31	(1.15-1.49)	1.60	(1.42-1.81)

9- < 12	1.26	(1.18-1.35)	1.47	(1.35-1.60)	1.49	(1.33-1.67)	1.54	(1.37-1.72)

12- < 16	1.19	(1.11-1.27)	1.27	(1.17-1.38)	1.34	(1.20-1.49)	1.38	(1.24-1.54)

≥16	1.0		1.0		1.0		1.0	

Marital status								

Married	0.81	(0.78-0.84)	0.81	(0.78-0.85)	0.79	(0.74-0.85)	0.94	(0.87-1.02)

Separated/Divorced	1.05	(1.02-1.07)	0.90	(0.87-0.93)	1.10	(1.04-1.16)	1.17	(1.09-1.26)

Widowed	0.99	(0.93-1.06)	0.87	(0.81-0.92)	1.23	(1.10-1.38)	1.11	(1.01-1.23)

Single/Never married	1.0		1.0		1.0		1.0	

Currently working								

Yes	0.95	(0.92-0.97)	0.80	(0.77-0.83)	0.95	(0.90-1.00)	0.84	(0.80-0.89)

No	1.0		1.0		1.0		1.0	

Neighborhood-level variables								

Household income								

< $18, 879	0.99	(0.93-1.06)	0.97	(0.88-1.08)	0.98	(0.87-1.10)	0.92	(0.81-1.04)

$18, 879 - < $26, 094	1.00	(0.94-1.06)	1.02	(0.94-1.11)	0.92	(0.84-1.01)	0.95	(0.87-1.04)

$26, 094 - < $34, 583	0.99	(0.94-1.04)	0.99	(0.92-1.06)	0.98	(0.92-1.05)	0.97	(0.91-1.03)

≥$34, 583	1.0		1.0		1.0		1.0	

Percent adults with ≥ HS education ^b^								

< 57.8%	0.97	(0.92-1.03)	1.02	(0.93-1.10)	1.00	(0.90-1.12)	1.02	(0.92-1.13)

57.8% - < 67.5%	0.97	(0.92-1.02)	1.02	(0.95-1.10)	0.95	(0.86-1.04)	1.00	(0.92-1.09)

67.5% - < 77.3%	0.99	(0.95-1.04)	1.00	(0.94-1.07)	1.02	(0.95-1.09)	1.02	(0.96-1.09)

≥77.3%	1.0		1.0		1.0		1.0	

Percent adults with ≥ college graduation^b^								

< 5.8%	1.06	(1.01-1.12)	1.15	(1.07-1.24)	1.00	(0.92-1.10)	1.02	(0.94-1.12)

5.8 - < 10.3%	1.08	(1.03-1.13)	1.08	(1.01-1.16)	0.98	(0.90-1.07)	1.01	(0.94-1.10)

10.3 - < 17.1%	1.03	(0.99-1.08)	1.03	(0.97-1.10)	0.98	(0.91-1.05)	1.02	(0.96-1.09)

≥17.1%	1.0		1.0		1.0		1.0	

Percent households owner occupied								

< 43%	1.20	(1.14-1.26)	1.34	(1.25-1.44)	1.08	(0.99-1.17)	1.05	(0.96-1.14)

43 - < 63%	1.14	(1.08-1.19)	1.22	(1.15-1.31)	1.04	(0.97-1.12)	1.05	(0.99-1.12)

63 - < 80%	1.10	(1.05-1.16)	1.15	(1.08-1.23)	0.98	(0.92-1.04)	0.97	(0.92-1.03)

≥80%	1.0		1.0		1.0		1.0	

Median owner-occupied household value								

< $44, 300	0.91	(0.87-0.95)	0.88	(0.82-0.94)	0.95	(0.86-1.04)	1.07	(0.98-1.17)

$44, 300 - < $57, 300	0.94	(0.90-0.98)	0.92	(0.86-0.98)	0.98	(0.90-1.06)	0.99	(0.92-1.08)

$57, 300 - < $77, 400	0.97	(0.93-1.01)	0.98	(0.92-1.04)	1.03	(0.96-1.10)	1.05	(0.98-1.11)

≥$77, 400	1.0		1.0		1.0		1.0	

Percent households with interest, dividends, or net rental income								

< 8.0%	1.04	(0.98-1.09)	1.05	(0.96-1.13)	1.20	(1.08-1.33)	1.02	(0.91-1.13)

8.0 - < 14.7%	1.05	(1.00-1.11)	1.02	(0.95-1.10)	1.12	(1.03-1.21)	1.00	(0.92-1.08)

14.7 - < 24.1%	1.03	(0.98-1.08)	0.99	(0.93-1.06)	1.03	(0.97-1.09)	1.02	(0.97-1.08)

≥24.1%	1.0		1.0		1.0		1.0	

Percent employed								

< 49.5%	1.02	(0.98-1.07)	1.07	(1.00-1.15)	0.99	(0.91-1.08)	0.99	(0.91-1.07)

49.5% - < 56.9%	1.00	(0.95-1.04)	1.07	(1.00-1.15)	0.97	(0.90-1.04)	0.98	(0.91-1.05)

56.9% - < 64.1%	0.99	(0.95-1.03)	1.04	(0.98-1.11)	0.97	(0.92-1.04)	1.04	(0.98-1.09)

≥64.1%	1.0		1.0		1.0		1.0	

^a^Each race and gender-specific column represents a single Poisson regression model that includes all individual-level and all neighborhood-level characteristics listed in the Table as well as participant age and duration of residency in the current home as reported during the baseline interview

^b^Among adults age 25 and older

For comparison with the Black Women's Health Study (BWHS) [8], we conducted additional analyses among black females that excluded all former smokers as was done in the BWHS report. In the SCCS, the PR (95% CI) for smoking comparing > 20% v.5% neighborhood poverty was 1.17 (1.07-1.29) and in the BWHS, the odds ratio was 1.6 (1.5-1.8).

## Discussion

In this large sample of black and white adults, several measures representing decreased neighborhood advantage were associated with increased prevalence of cigarette smoking after adjustment for individual-level SES although the associations varied to some extent by race and gender. The overall associations between smoking and neighborhood-level SES in our study were consistent with those among black women enrolled in the large BWHS [8] and the CARDIA study of young black and white adults [5] as well as from other smaller US-based studies [4,7,13]. Collectively, our findings as well as those from other studies point to an overall modest but significant negative effect of lower neighborhood-level SES on cigarette smoking after adjustment for individual-level SES measures such as education and income that are known to be associated with smoking behavior. Much speculation has been made for the potential mechanisms behind these effects and include factors related to neighborhood context (such as social norms, psychosocial stress, and exposure to tobacco advertising) as well as potential influences of individuals and their behavior on other individuals within neighborhoods, sometimes called the contagion perspective [4,5,8].

Our study fills a sizeable gap in the literature by examining smoking in relation to neighborhood-level characteristics in a population of both black and white men and women over a wide age range where neighborhood poverty was common, an especially important population to study because of the high prevalence of cigarette smoking [3]. Interventions to prevent smoking initiation and increase smoking cessation are desperately needed to reduce the morbidity and mortality associated with smoking, and the results from this study as well as others that have examined neighborhood characteristics in relation to smoking indicate that the development of interventions that target high-risk neighborhoods may be beneficial. Further, this work indicates that these interventions may be tailored to specific subgroups of race or gender that might be especially affected by aspects of the area in which they reside.

With respect to differences observed by race, individual-level household income and educational attainment were similar between black and white SCCS participants, but blacks were considerably more likely than whites to live in more disadvantaged neighborhoods with more poverty and lower percentages of highly educated and professional residents. This type of residential segregation has been described previously [26] and indicates that further investigation is warranted into as-yet unmeasured aspects of neighborhood settings that may differentially affect smoking behavior such as racial differences in social support and cultural norms. In the SCCS population, there was a significant inverse association between neighborhood-level percentage of adults with a college education and smoking behavior among blacks but not whites. The opposite was observed in the CARDIA study [5] and no association was seen for this measure among black females in the BWHS [8]; these inconsistencies may be related to different distributions of individual-level and neighborhood-level education levels in the SCCS compared with other studies.

The lack of association between neighborhood SES and smoking prevalence among white women in the SCCS was noTable in this analysis. The individual measures of income and education were most strongly associated with smoking in white women, and these effects may have overwhelmed small effects of neighborhood SES in the statistical models. Unmeasured aspects of both the individual and neighborhood environment are also possible explanations for differences in white women from other groups such as stress, peer behavior, and social support for quitting.

Additionally, we found some evidence that the association between smoking and living in a disadvantaged neighborhood (as measured by the neighborhood advantage summary score) was greater in individuals with higher rather than lower individual-level household income. Diez Roux et al. observed a similar effect among blacks (combined over gender) in their analysis of young adults in the CARDIA study [5]. These findings are contrary to the often-hypothesized notion that individuals of lower SES are more susceptible to the negative effects of living in disadvantaged neighborhoods due to increased pressure to engage in negative-health behaviors or lack of resources and positive supports. These results suggest that neighborhood pressures may be stronger in individuals of higher individual SES, but future work to determine why and how individual-level factors such as income might differentially affect the impact of neighborhood context on smoking behavior is needed.

Despite general trends indicating an inverse association between neighborhood SES and smoking behavior, many inconsistencies exist in the current literature for specific SES characteristics, particularly across race and gender lines. Some of the inconsistencies across studies may be related to the specification of the smoking measure. In our analysis, we compared current smokers to non-current smokers, a group which consisted of both former and never smokers; the same measure was used in at least two other studies [4,5]. Other metrics have included comparisons of ever v. never smokers and current v. former smokers [7] and current v. never smokers after excluding former smokers [8]. Inconsistencies across studies could also be related to the use of census tracts versus census block groups, but comparisons of effects using these two geographic entities showed little variation in at least one comparison study [5].

A major strength of this investigation was the utilization of the SCCS cohort which includes large numbers of black and white participants of generally similar individual-level socioeconomic and geographic situation, enhancing comparability between race and gender groups. While a majority of SCCS participants are of low SES, the large sample also includes sizeable numbers of individuals of higher SES allowing for robust comparisons across the spectrum of education and income levels. Additionally, there was sufficient overlap in this study population of individuals across all categories of individual-level and neighborhood-level SES to assess these measures together. We also used robust modeling techniques which allowed for the assessment of the relative contributions of individual- and neighborhood-level characteristics as well as the estimation of more accurate standard errors than those produced using standard modeling techniques. Limitations should also be considered. First, the SCCS is not a strictly population-based sample; because of the unique recruitment of participants through southeastern CHCs and the resulting high proportion of low SES and other factors (such as high smoking prevalence), the results observed here may not be generalizable to the entire US population. However, it should be emphasized that while generalizability is a limitation, the SCCS design uniquely increases internal validity when making comparisons of effects across race groups. A second limitation of this study is that the cross-sectional nature of the data limits our ability to make temporal inferences about the association between individual and neighborhood-level characteristics and current cigarette smoking. However, as has previously been observed, current neighborhood characteristics may exert influence on smoking quitting patterns even if it did not influence its initiation [27]. Additionally, the use of census block groups as proxies for neighborhoods requires assumptions which could not be evaluated including that census block group characteristics uniformly affect all individuals within the group and that block group boundaries adequately represent an individual's neighborhood. A final limitation related to the use of the 2000 census data is that SES characteristics within block groups may have changed over the 2002-2009 SCCS enrollment period although it should be noted that half of the cohort was enrolled by 2004 and only 14% enrolled after 2007.

## Conclusions

In summary, in this large sample of black and white individuals living in the southeastern United States, we observed modest but significant associations between several measures of neighborhood-level SES and current smoking behavior. These results can be used to inform the development and testing of a comprehensive framework that takes into account the potentially differing influences of individual-level and neighborhood-level SES-related factors affecting smoking behavior. Ultimately a greater understanding of these relationships can be used to develop smoking cessation initiatives targeted to both individuals and neighborhoods at the highest risk of the negative health effects of cigarette smoking.

## Abbreviations

CHC: Community Health Center; CI: Confidence interval; PR: Prevalence ratio; SCCS: Southern Community Cohort Study; SES: Socioeconomic status

## Competing interests

The authors declare that they have no competing interests.

## Authors' contributions

All authors conceived the study. SSC supervised the study, supervised the statistical analyses, and led the writing. MTT performed the data linkage to the US Census. JSS and MTT performed the statistical analyses. All authors contributed critical revisions for content to the manuscript. All authors read and approved the final manuscript.

## Pre-publication history

The pre-publication history for this paper can be accessed here:

http://www.biomedcentral.com/1471-2458/11/877/prepub

## References

[B1] US Department of Health and Human Services The health consequences of smoking: a report of the surgeon general 2004 Atlanta: U.S. Department of Health and Human Services, Centers for Disease Control and Prevention, National Center for Chronic Disease Prevention and Health Promotion, Office on Smoking and Health

[B2] Heyman KM Barnes PM Schiller JS Early release of selected estimates based on data from the January-September 2009 National Health Interview Survey 2010 Hyattsville, MD: National Center for Health Statistics http://www.cdc.gov/nchs/nhis.htm

[B3] Cokkinides V Bandi P McMahon C Jemal A Glynn T Ward E Tobacco control in the United States--recent progress and opportunities CA Cancer J Clin 2009 59 6 352 365 10.3322/caac.20037 19897839

[B4] Ross CE Walking, exercising, and smoking: does neighborhood matter? Soc Sci Med 2000 51 2 265 274 10.1016/S0277-9536(99)00451-7 10832573

[B5] Diez Roux AV Merkin SS Hannan P Jacobs DR Kiefe CI Area characteristics, individual-level socioeconomic indicators, and smoking in young adults: the coronary artery disease risk development in young adults study Am J Epidemiol 2003 157 4 315 326 10.1093/aje/kwf207 12578802

[B6] Shohaimi S Luben R Wareham N Day N Bingham S Welch A Oakes S Khaw KT Residential area deprivation predicts smoking habit independently of individual educational level and occupational social class. A cross sectional study in the Norfolk cohort of the European Investigation into Cancer (EPIC-Norfolk) J Epidemiol Community Health 2003 57 4 270 276 10.1136/jech.57.4.270 12646543 PMC1732421

[B7] Tseng M Yeatts K Millikan R Newman B Area-level characteristics and smoking in women Am J Public Health 2001 91 11 1847 1850 10.2105/AJPH.91.11.1847 11684614 PMC1446889

[B8] Datta GD Subramanian SV Colditz GA Kawachi I Palmer JR Rosenberg L Individual, neighborhood, and state-level predictors of smoking among US Black women: a multilevel analysis Soc Sci Med 2006 63 4 1034 1044 10.1016/j.socscimed.2006.03.010 16650514

[B9] Sundquist J Malmstrom M Johansson SE Cardiovascular risk factors and the neighbourhood environment: a multilevel analysis Int J Epidemiol 1999 28 5 841 845 10.1093/ije/28.5.841 10597980

[B10] Kandula NR Wen M Jacobs EA Lauderdale DS Association between neighborhood context and smoking prevalence among Asian Americans Am J Public Health 2009 99 5 885 892 10.2105/AJPH.2007.131854 19299683 PMC2667865

[B11] Adams RJ Howard N Tucker G Appleton S Taylor AW Chittleborough C Gill T Ruffin RE Wilson DH Effects of area deprivation on health risks and outcomes: a multilevel, cross-sectional, Australian population study International journal of public health 2009 54 3 183 192 10.1007/s00038-009-7113-x 19214382

[B12] Luke D Esmundo E Bloom Y Smoke signs: patterns of tobacco billboard advertising in a metropolitan region Tob Control 2000 9 1 16 23 10.1136/tc.9.1.16 10691754 PMC1748288

[B13] Diez-Roux AV Nieto FJ Muntaner C Tyroler HA Comstock GW Shahar E Cooper LS Watson RL Szklo M Neighborhood environments and coronary heart disease: a multilevel analysis Am J Epidemiol 1997 146 1 48 63 9215223 10.1093/oxfordjournals.aje.a009191

[B14] Signorello LB Hargreaves MK Blot WJ The Southern Community Cohort Study: investigating health disparities J Health Care Poor Underserved 2010 21 1 Suppl 26 37 20173283 10.1353/hpu.0.0245 PMC2940058

[B15] Signorello LB Hargreaves MK Steinwandel MD Zheng W Cai Q Schlundt DG Buchowski MS Arnold CW McLaughlin JK Blot WJ Southern community cohort study: establishing a cohort to investigate health disparities J Natl Med Assoc 2005 97 7 972 979 16080667 PMC2569308

[B16] Hargreaves MK Arnold C Blot WJ Satcher D, Pamies R Community health centers: their role in the treatment of minorities and in health disparities research Multicultural Medicine and Health Disparities 2006 New York: McGraw-Hill 485 494

[B17] Southern Community Cohort Study (SCCS) http://www.southerncommunitystudy.org/

[B18] ESRI Streetmap USA in ESRI Data & Maps [machine-readable datafiles] 2005

[B19] US Census Bureau 2008 TIGER/Line^® ^Shapefiles [machine-readable data files] 2008

[B20] Census 2000 summary file 3 technical documentation prepared by the U.S. Census Bureau 2002

[B21] Diez-Roux AV Kiefe CI Jacobs DR Haan M Jackson SA Nieto FJ Paton CC Schulz R Area characteristics and individual-level socioeconomic position indicators in three population-based epidemiologic studies Ann Epidemiol 2001 11 6 395 405 10.1016/S1047-2797(01)00221-6 11454499

[B22] Zeger SL Liang KY Longitudinal data analysis for discrete and continuous outcomes Biometrics 1986 42 1 121 130 10.2307/2531248 3719049

[B23] McNutt LA Wu C Xue X Hafner JP Estimating the relative risk in cohort studies and clinical trials of common outcomes Am J Epidemiol 2003 157 10 940 943 10.1093/aje/kwg074 12746247

[B24] Zou G A modified poisson regression approach to prospective studies with binary data Am J Epidemiol 2004 159 7 702 706 10.1093/aje/kwh090 15033648

[B25] Spiegelman D Hertzmark E Easy SAS calculations for risk or prevalence ratios and differences Am J Epidemiol 2005 162 3 199 200 10.1093/aje/kwi188 15987728

[B26] Iceland J Sharpe C Steinmetz E Class differences in African American residential patterns in US metropolitan areas: 1990-2000 Soc Sci Res 2005 34 1 252 266 10.1016/j.ssresearch.2004.02.001

[B27] Giskes K van Lenthe FJ Turrell G Brug J Mackenbach JP Smokers living in deprived areas are less likely to quit: a longitudinal follow-up Tob Control 2006 15 6 485 488 10.1136/tc.2006.015750 17130379 PMC2563671

